# Supplementary material for: Rapid Determination of Mixed Pesticide Residues on Apple Surfaces by Surface-Enhanced Raman Spectroscopy
Source: Foods. 2022 Apr 10;11(8):1089. doi: 10.3390/foods11081089 (PMC9031303; doi:10.3390/foods11081089)
Supplement: Supplementary file 1 [file foods-11-01089-s001.zip › foods-1645199-supplementary.pdf]

Rapid Determination of Mixed Pesticide Residues on Apple Surfaces by Surface-Enhanced Raman Spectroscopy

Luyao Wang [1], Pei Ma [1], Hui Chen [1], Min Chang [1], Ping Lu [1], Ning Chen [1], Yanbing Yuan [1], Nan Chen [2] and Xuedian Zhang [1,3,*]

1. Key Laboratory of Optical Technology and Instrument for Medicine, Ministry of Education, College of Optical-Electrical and Computer Engineering, University of Shanghai for Science and Technology, Shanghai 200093, China; wangluyao0106@126.com (L.W.); peima@usst.edu.cn (P.M.); chenhui@usst.edu.cn (H.C.); changmin@usst.edu.cn (M.C.); lu945632952@163.com (P.L.); chenning103420@163.com (N.C.); 15054596156@163.com (Y.Y.)
2. School of Electrical Engineering, Nantong University, Nantong 226019, China; ntu_chennan@ntu.edu.cn
3. Shanghai Institute of Intelligent Science and Technology, Tongji University, Shanghai 200092, China

* Correspondence: obmmd_zxd@163.com

The Calculation of LOD and LOQ:

LOD refers to the limit of detection, which was calculated as the International Union of Pure and Applied Chemistry (IUPAC) recommendation. Consequently, the equation for determination of LOD is LOD = 3σ/S [1,2]. Meanwhile, the limit of quantification (LOQ) can be calculated by the equation LOQ = 10σ/S. Herein, σ represents the standard deviation of blank signal. S represents the sensitivity or slope of the calibration curve, which is determined by Δintensity/Δconcentration.

In our study, the value of σ was 125 (a.u.) calculated based on the intensities of 20 SERS signals at 675 cm^{-1}. The average SERS intensity at 675 cm^{-1} corresponding to CPF with a concentration of 2.85×10^{-9} mol L^{-1} was measured to be 835 (a.u.), which indicated the sensitivity S=292.98×10^{9}. Thus, the LOD and LOQ for CPF were calculated to be 1.28×10^{-9} mol L^{-1} and 4.27×10^{-9} mol L^{-1}. Similarly, LOD and LOQ for 2,4-D were determined to be 2.47×10^{-10} mol L^{-1} and 8.23×10^{-10} mol L^{-1}.

Table S1. Standard deviation calculation result of CPF standard solution and apple surface sample.

CPF	Concentrations (mg L^{-1})	1000	100	10	1	0.1	0.01	0.001
Standard solution	RSD (%)	2.51	3.39	3.57	4.76	5.35	6.89	8.95
Sample solution	RSD (%)	3.36	3.94	4.23	5.25	6.27	7.58	8.98

Table S2. Standard deviation calculation result of 2,4-D standard solution and apple surface sample

2,4-D	Concentrations (mg L^{-1})	1000	100	10	1	0.1	0.01	0.001	0.0001
Standard solution	RSD (%)	2.91	3.62	3.95	4.79	5.62	6.73	7.58	8.78
Sample solution	RSD (%)	3.27	4.15	4.48	5.25	6.14	7.63	8.57	8.99

References

1. Fang, H.; Zhang, X.; Zhang, S.J.; Liu, L.; Zhao, Y.M.; Xu, H.J. Ultrasensitive and quantitative detection of paraquat on fruits skins via surface-enhanced Raman spectroscopy. *Sens. Actuators B Chem*. **2015**, *213*, 452–456.
2. Chen, H.; Luo, C.; Xing, L.; Guo, H.; Ma, P.; Zhang, X.; Zeng, L.; Sui, M. Simultaneous and ultra-sensitive SERS detection of SLPI and IL-18 for the assessment of donor kidney quality using black phosphorus/gold nanohybrids. *Opt. Express* **2022**, *30*, 1452–1465.
